# PHOSPHORYLATION OF ULK1 AT S555 IS REQUIRED FOR METABOLIC ADAPTATIONS TO CALORIC RESTRICTION

**DOI:** 10.1093/geroni/igac059.1668

**Published:** 2022-12-20

**Authors:** Joshua Drake, Anna Nichenko, Orion Wiloughby, Matt Brisendine, Garrett Hays, Grace DiGirolamo, Zach Weingrad, Ryan McMillan

**Affiliations:** Virginia Polytechnic Institute and State University, Blacksburg, Virginia, United States; Virginia Polytechnic Institute and State University, Blacksburg, Virginia, United States; Virginia Polytechnic Institute and State University, Blacksburg, Virginia, United States; Virginia Polytechnic Institute and State University, Blacksburg, Virginia, United States; Virginia Polytechnic Institute and State University, Blacksburg, Virginia, United States; Virginia Polytechnic Institute and State University, Blacksburg, Virginia, United States; Virginia Polytechnic Institute and State University, Blacksburg, Virginia, United States; Virginia Polytechnic Institute and State University, Blacksburg, Virginia, United States

## Abstract

Unc-51 Like Autophagy Activating Kinase 1 (Ulk1) is responsible for initiating selective degradation of damaged/dysfunctional mitochondria (mitophagy) once phosphorylated at S555 in response to energetic stress. Mitophagy is integral for mitochondrial health and Ulk1 has been implicated to be important for metabolic adaptation to exercise. Caloric restriction (CR), which extends lifespan and healthspan, has profound metabolic benefits, including improved mitochondrial health. However, the contribution of Ulk1 in adaptation to CR is unknown. To decipher a functional role of Ulk1(S555) in adaptations to CR we used CRISPR-Cas9 generated, loss-of-function Ulk1(S555A) mice, in which Ulk1 cannot be phosphorylated at S555. 6-month-old, male and female homozygous Ulk1(S555A) mice and C57BL6/J (wild type, WT) mice were placed on a 40% CR diet for 8 weeks. Body mass in both male and female Ulk1(S555A) and WT mice was reduced with CR (p < 0.001), however female Ulk1(S555A) were heavier than their WT counterparts (p=0.02). Via nuclear magnetic resonance (NMR), male and female Ulk1(S555A) mice did not lose fat mass during CR. In addition, periovarian (female) and epididymal (male) fat mass was greater in Ulk1(S555A) compared to WT mice post-CR (p < 0.001). Furthermore, fasting blood glucose increased in male and female Ulk1(S555A) post-CR (p < 0.0001), suggesting altered substrate metabolism. In support of this notion, glucose oxidation in both quadriceps muscle and liver of male mice increased in WT following CR but not in Ulk1(S555A) mice (interaction effect p< 0.002). In sum, these data suggest that phosphorylation of Ulk1 at S555 is required for metabolic adaptations to CR.

